# Moment-to-Moment Transfer of Positive Emotions in Daily Life Predicts Future Course of Depression in Both General Population and Patient Samples

**DOI:** 10.1371/journal.pone.0075655

**Published:** 2013-09-23

**Authors:** Petra Höhn, Claudia Menne-Lothmann, Frenk Peeters, Nancy A. Nicolson, Nele Jacobs, Catherine Derom, Evert Thiery, Jim van Os, Marieke Wichers

**Affiliations:** 1 Department of Psychiatry and Psychology, School for Mental Health and Neuroscience, Maastricht University Medical Centre, Maastricht, The Netherlands; 2 Faculty of Psychology, Open University, Heerlen, The Netherlands; 3 Centre of Human Genetics, University Hospitals, Leuven, & Department of Human Genetics, KU Leuven, Leuven, Belgium; 4 Department of Neurology, Ghent University Hospital, Ghent University, Ghent, Belgium; 5 Department of Psychosis Studies, King’s College London, King’s Health Partners, Institute of Psychiatry, London, United Kingdom; University of California San Diego, United States of America

## Abstract

**Objective:**

Positive affect (PA) is closely linked to prevention of, and recovery from, depression. Previous studies have investigated PA *reactivity* to pleasant situations with respect to its protective properties in relation to mood disorder. The purpose of this study was to examine, and replicate, whether moment-to-moment transfer of PA in daily life (PA *persistence*) is relevant to the prediction of future course of depression.

**Method:**

Individuals from three different studies (one general population sample (n=540), and two patient samples (n=43 and n=50) with matching controls (n=39 and n=21, respectively)) participated in an Experience Sampling Method (ESM) study. Time-lagged multilevel analyses were used to assess the degree of transfer (or persistence) of momentary positive affective states over time, in relation to naturalistic outcome (study 1) or treatment outcome (studies 2 and 3). Depressive symptoms were measured using the Symptom Checklist (SCL-90R) in sample 1 and the Hamilton Depression Rating Scale (HDRS) in samples 2 and 3.

**Results:**

In study 1, participants with greater momentary PA persistence were less likely to show depressive symptoms at follow-up. In study 2, patients were more likely to respond to treatment if they displayed greater momentary PA persistence, particularly in those with recurrent depression. In study 3, patients with greater momentary PA persistence were similarly more likely to respond to treatment, especially when treated with imipramine rather than placebo.

**Conclusion:**

The ability to transfer PA from one moment to the next is an important factor in the prevention of and recovery from depressive symptoms. Patients with recurrent depression and those who receive antidepressants rather than placebo may benefit most from this effect. The results suggest that treatment-induced improvement in depression is mediated by increased levels of momentary transfer of PA in daily life, acquisition of which may be contingent on duration of exposure to depressive experience.

## Introduction

There is considerable evidence showing that being able to experience positive affect (PA) has beneficial effects on mental health, particularly in depression. Studies have shown that PA works by undoing the effects of stress [1-5]. In addition, experience of PA decreases the likelihood of follow-up depressive symptoms [2,3,6], and facilitates remission from a depressive episode [7-10].

Positive and negative affective states do not represent two extremes of the same dimension [11]. Rather, they constitute distinct dimensions of emotional experience, with distinct adaptive functions, mediation of which takes place in different brain systems [12,13]. Where negative affect (NA) may inform the person of imminent threat (with respect to life, future, or hierarchical position in the group), PA has the function of engaging the person with the environment and other individuals in social networks [13,14]. This function is reflected in the fact that positive affective states have an inverted U-shaped curve over the day [15], suggesting that most PA is experienced in the middle of the day when there are more opportunities to encounter pleasant situations. The broaden-and-build theory [14] suggests that positive emotions have a direct impact on attentional awareness, making us more open to experience and contact. This theory also suggests that positive emotions facilitate the building of social networks, resulting in long-term well-being and social connectedness. Garland and colleagues [16] characterized this as the positive spiral of positive emotions: increased social openness and positive behavioral repertoire will, in turn, again lead to new experiences of positive emotions. Taken together, PA appears to significantly contribute to mental health.

There are different ways to acquire PA. One is the experience of PA in response to natural rewards. Reactivity to rewarding situations has been examined both experimentally and prospectively in the flow of daily life. Experimental studies expose individuals to pleasant images in the laboratory or use computer tasks in which participants can earn certain monetary rewards [17-20]. Other studies prospectively measured appraisals of situations and affective states, and examined reactivity to pleasant situations in the flow of daily life [2,8,9,21,22].

Second, retention of earlier acquired PA contributes to subsequent positive emotional experience over time. Only few studies have tried to examine this phenomenon in relation to depressive symptoms. Heller and colleagues [23] found that depressed patients have difficulty sustaining activity in brain areas that are responsible for PA and reward, suggesting reduced PA persistence in depression. Two other experimental studies measured persistence of PA and NA in the laboratory, during a 20-minute family interaction task between adolescents and their parents, focused at resolving an area of conflict. The conversations were videotaped and emotional fluctuations were measured by rating the verbal and non-verbal behavior of healthy and depressed adolescents during this interaction. Results showed that, compared to healthy participants, depressed adolescents were more emotionally resistant; that is, showed less emotional reactivity to changing circumstances [24]. In healthy adolescents, this emotional resistance to both positive and negative change predicted follow-up onset of major depression [25]. To date, only one study has measured persistence of PA in daily life. This study compared the extent to which individuals retained PA over time in a group of 53 depressed adults compared to a group of 53 healthy controls [26]. No differences between groups were found.

Taken together, only few studies have prospectively examined PA persistence in relation to depression. These studies used different designs and yielded dissimilar conclusions. There is an urgent need for replication of findings as true replication of studies across different designs is challenging. Therefore, in the present study we explored PA persistence in three different samples by examining moment-to-moment transfer of positive affect in the flow of real life. Studies that measure PA persistence prospectively in the flow of daily life are particularly scarce. To our knowledge, to date no study has examined prospectively, in the flow of daily life, how PA persistence –conceptualized as moment-to-moment transfer of PA- predicts future course of depression. For this purpose, we used the Experience Sampling Method (ESM) [27-30].

ESM is an assessment technique that allows prospective tracking of momentary experience in daily life. The advantage of this technique is that subtle moment-to-moment transitions in affective states are captured and assessed in the moment, with minimal retrospection and cognitive reinterpretation. Furthermore, ESM has strong ecological validity, as measurements are performed in everyday life [31].

The aim of this study was to examine and replicate, in three different samples, whether moment-to-moment transfer of PA predicts future course of depressive symptoms. First, we examined in a general female population sample whether moment-to-moment transfer of PA would be associated with follow-up symptoms of depression. Second, we examined in a sample of depressed patients whether differences in moment-to-moment transfer of PA would be associated with differences in treatment response. In addition, in this sample we were able to examine whether this effect would differ for patients with first episode versus recurrent depression, as previous work suggests that this is an important moderator of treatment response [32]. Finally, in a third sample we investigated again whether differences in moment-to-moment transfer of PA would be associated with differences in treatment response in depressed patients. The data of this sample, however, were derived from a randomized controlled trial in which depressed participants were allocated to imipramine or placebo treatment. Therefore, this sample allowed for an additional moderation analysis comparing active pharmacological treatment with placebo. For both patient samples, matched healthy controls were available to additionally compare effects as a function of clinical status.

## Methods

**Figure 1:**
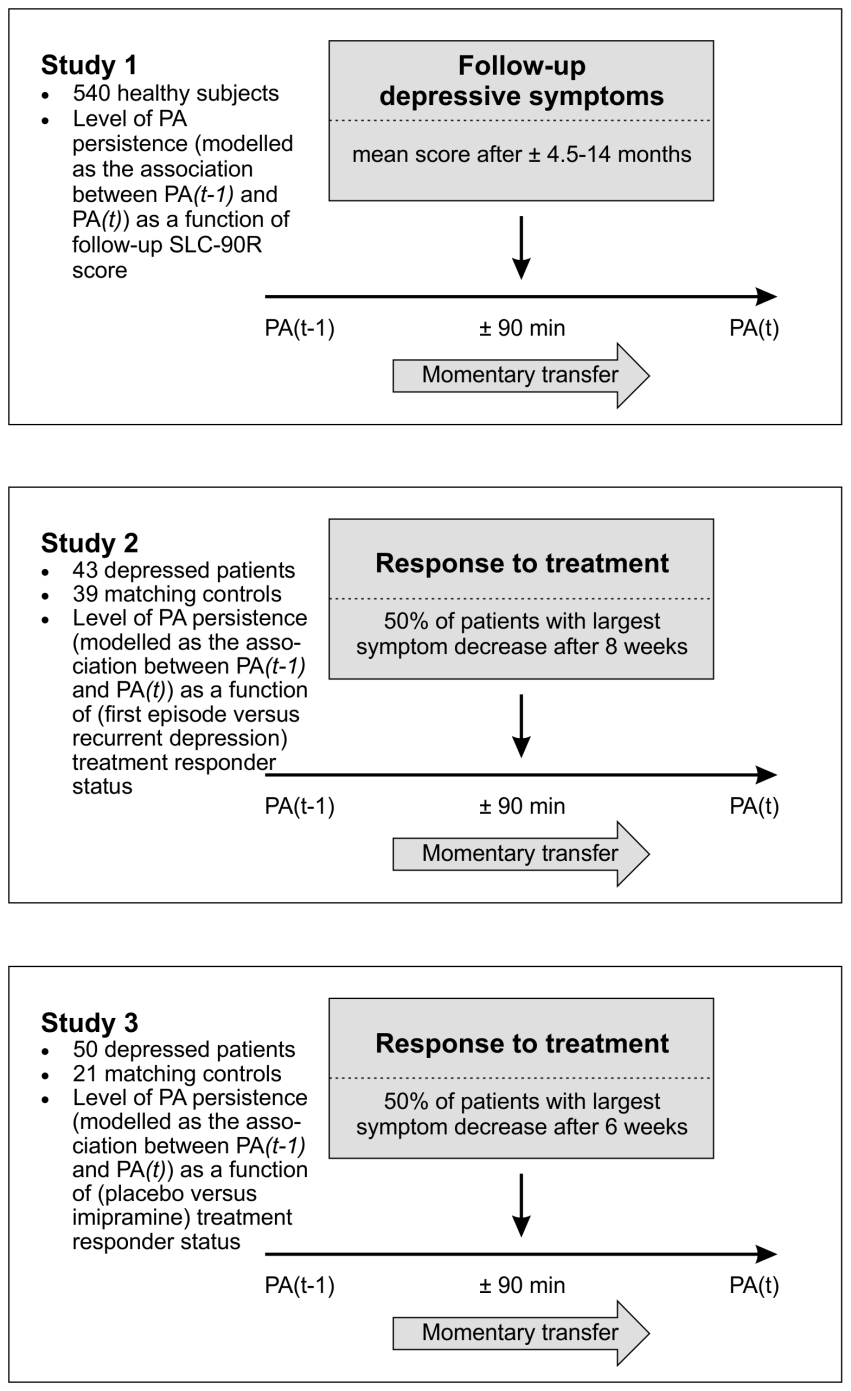
Schematic plan of analyses for study 1, 2, and 3. Effect of momentary transfer of positive affect (PA) from (*t-1*) to (*t*) approximately 90 minutes later, moderated by (1) follow-up depressive symptoms, defined by mean SCL-90R score at four follow-up assessments spread over 14 months, (2) response to treatment with imipramine/placebo, defined by HDRS score after six weeks, and (3) response to treatment at first episode/recurrent depression, defined by HDRS score over eight weeks.

Three separate studies were carried out to answer the above research questions. For a graphical overview of the design and research questions of each study, see Figure 1. The same ESM was employed across the three studies.

### Ethics statement

Study 1 was approved by the Committee of Medical Ethics of the University of Leuven, Belgium. Studies 2 and 3 were approved by the Medisch-Ethische Toetsingscommissie of the Academic Hospital in Maastricht/Maastricht University (METC azM/UM), the Netherlands. All participants provided written informed consent.

### Experience sampling method

ESM is a structured diary technique to assess individuals in their daily living environment; it has been extensively validated for the use of momentary affect measurements [27,29,30]. In the present three studies, participants received a digital wristwatch and a set of ESM self-assessment forms collated in a booklet for each day. The wristwatch was programmed to emit a signal (‘beep’) at an unpredictable moment in each of ten 90-min time blocks between 07:30 and 22:30, on five or six consecutive days, depending on the study. After each beep, participants were asked to fill out the ESM self-assessment forms, collecting reports of mood. All self-assessments were rated on seven-point Likert scales. Trained research assistants with ample experience in momentary assessment techniques explained the ESM procedure to the participants during an initial briefing session and a practice form was completed to confirm that all were able to understand the scales. Subjects could call a telephone number in case they had questions or problems during the ESM sampling period. Participants were instructed to complete their reports immediately after the beep, thus minimizing memory distortion, and to record the time at which they completed the form. In order to verify compliance, the reported completion time was compared with the actual time of the beep. All reports not filled in within 15 minutes after the beep were excluded from the analysis, as previous work has shown that reports completed after this interval are less reliable and consequently less valid [27]. In addition, participants with fewer than 17 out of 50 (study 1), or 20 out of 60 (studies 2 and 3) valid reports were excluded from the analysis, as previous work has shown that measures of individuals with less than 30% of completed reports are less reliable [27].

## Study 1

### Participants

We investigated in a sample of 621 participants from the East-Flanders Prospective Twin Survey in Belgium –a general population sample that has been followed prospectively to study gene–environment interactions in affective disorders [33]- whether people with greater momentary transfer of PA are less likely to develop follow-up depressive symptoms. Although most subjects were twins, the current study did not require twin methodology.

Of the 621 participants, 610 completed the ESM measurements. Thirty-one were subsequently excluded due to inadequate ESM compliance and 39 due to missing follow-up data on depressive symptoms, resulting in a final sample of 540 participants. The mean number of completed ESM reports was 38.0 (SD 6.6; range 17-50). Participants were white and of Belgian origin; mean age was 27.6 (SD 7.8; range 18-61). Thirty-four percent of participants were married, 35% had completed secondary education, and 63% had college or university degrees. The majority (60%) was employed.

### Study design

ESM took place at baseline (T0) for five consecutive days. Depressive symptoms were assessed at (T0) and again at each of the four follow-up assessments (T1-T4), conducted at intervals of three to four months.

### Measurements

#### Affective states

PA was assessed at each beep during the ESM procedure. Since some analyses had to be controlled for level of NA, this was assessed in a similar fashion as PA. Participants rated ten momentary affective states on 7-point Likert scales (1: not at all to 7: very). Our choice of the ESM affect items was guided by the PANAS questionnaire [34] and by results of previous ESM studies (selecting items with high loadings on NA and PA latent factors and sufficient within-person variability). Factor analysis, using principal component analysis with oblique rotation, identified two mood factors with eigenvalue >1 [8]. Ratings for the items ‘cheerful’, ‘content’, ‘energetic’, and ‘enthusiastic’ –weighted for factor loadings- were averaged to form the PA scale. The weighted average of ratings for ‘insecure’, ‘lonely’, ‘anxious’, ‘low’, ‘guilty’, and ‘suspicious’ formed the NA scale.

#### Depressive symptoms

All participants completed the Symptom Checklist (SCL-90R) [35], which yielded prospective measures of depressive symptoms over time. As a measure of follow-up depressive symptomatology, we used the mean of the SCL-90R scores over time points T1 through T4. In order to improve normality, the SCL-90R depression score was log-transformed.

### Statistical analysis

ESM data have a hierarchical structure with multiple observations (level 1) clustered within participants (level 2). Multilevel analysis takes the variability associated with each level of nesting into account [36]. Multilevel mixed regression analyses, using the *xtmixed* command in Stata 11.1 (StataCorp, 2009), were applied to the data. To obtain a regression model that specifically tests within-subject effects, we centered the variables PA and NA around the individual mean. The model estimated the extent to which PA level at moment (*t-1*) (independent variable) predicted PA level at the next moment (*t*), approximately 90 minutes later on the same day (dependent variable), reflecting the degree of momentary PA transfer or PA persistence. By fitting an interaction term between PA at (*t-1*) and mean SCL-90R scores at T1-T4 in the model of PA at (*t*), we tested whether PA persistence differed according to level of depressive symptoms at follow-up, corrected for baseline depressive symptoms (Figure 1). This model thus effectively tests whether the association between baseline PA and follow-up SCL-90R scores is contingent on the level of PA persistence. We controlled for level of NA at moment (*t*) to ensure that any effect of momentary transfer of PA was in fact independent from level of NA. All effect sizes were standardized and calculated from the model containing the interaction with the Stata *lincom* command.

### Results

**Figure 2:**
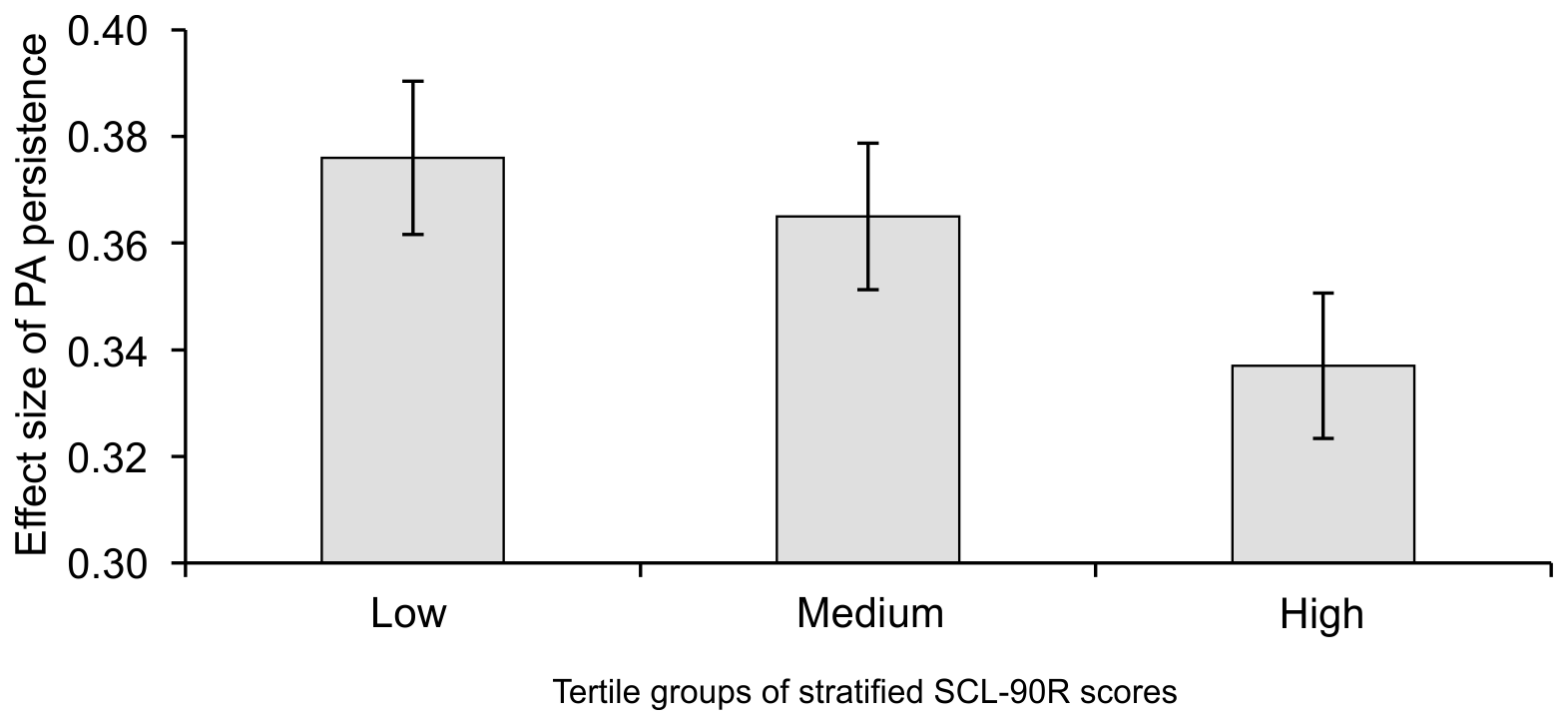
Degree of momentary PA persistence at baseline as a function of follow-up depressive symptom severity. Bars represent the association between PA at (*t-1*) and PA at (*t*) (level of momentary transfer of positive affect (PA) from (*t-1*) to (*t*) approximately 90 minutes, or level of PA persistence), stratified by SCL-90R severity level. Error bars represent one standard error of the mean.

Overall, level of PA at a given beep was a significant predictor of level of PA at the next beep (β=.370, SE=.007, *p*<.001). Furthermore, in the model of PA at (*t*) at baseline, there was a significant negative interaction between PA at (*t-1*) and level of follow-up depressive symptoms (β interaction=-.015, SE=.007, *p*<.05). Participants with greater momentary transfer of PA showed lower levels of depressive symptoms at follow-up. A dose-response association was apparent. Standardized effect sizes, stratified by the tertile groups of follow-up depressive symptoms are displayed in Figure 2.

## Study 2

### Participants

The second sample consisted of 47 depressed patients recruited at a university-affiliated outpatient mental health center in Maastricht, the Netherlands [37]. Inclusion criteria were a DSM-IV diagnosis of major depressive disorder (MDD), age between 18 and 65 years, and a baseline score of ≥18 on the 17-item Hamilton Depression Rating Scale (HDRS) [38]. Exclusion criteria included bipolar disorder, substance abuse, psychotic symptoms, and insufficient Dutch language proficiency. Moreover, none of the participants was taking antidepressants or other psychotropic medication, other than low dose benzodiazepines. Fifty-five healthy participants, matched as a group to the patient sample for sex and age, were recruited from available research pools and advertisements in a local newspaper. Additional exclusion criteria for the healthy participants were a lifetime history of any DSM-IV Axis I disorder or any inpatient treatment for an Axis I psychiatric disorder in a first-degree relative. Participants who completed the study received a 30 euro gift certificate.

In the patient group, one participant was excluded due to insufficient valid ESM reports, and three patients had missing HDRS scores. Of 55 potential controls who were screened, 16 did not meet the inclusion criteria. The mean number of completed ESM reports was 49.6 (SD 7.7; range 20-60). The final sample consisted of 82 participants: 43 depressed and 39 matched healthy participants (Table 1). The 25 patients who were depressed for the first time were categorized as first episode patients, of which 12 were classified as responders to treatment. The 18 patients with one or more prior episodes of depression were categorized as recurrent episode patients, of which nine were classified as responders to treatment.

**Table 1:** Demographic data for depressed patients and healthy controls.

	Depressed patients	Healthy controls
N	43	39
% women	58	58
Mean age (SD)	39.8 (11.1)	44.3 (11.6)
- range	20-58	22-62
% married	86	59
% secondary education	56	89
% employed	37	79

SD = standard deviation

### Study design

All participants (including the healthy controls) underwent ESM at baseline for six consecutive days. Immediately after the ESM sampling week, the depressed patients entered a naturalistic treatment phase (as described in [39]), during which they received a combination of pharmacotherapy and supportive psychotherapy. MDD treatment was administered according to standard guidelines [40]. Typically, sessions were weekly and decreased in frequency following agreement between therapist and participant. Therapies at the treatment center typically lasted between 15 and 20 therapy sessions. Participants were prescribed serotonergic antidepressants in flexible dosage depending on participants’ response and tolerance. In case of non-response, medication was switched to venlafaxine or a tricyclic agent, and lithium was added in case of enduring non-response. No participants received MAO inhibitors or electroconvulsive therapy (ECT). In order to assess the course of depression, the HDRS was administered at screening and thereafter at baseline, week four, and week eight.

### Measurements

#### Affective states

The ESM affect items were selected according to the method described in study 1. Factor analysis, using principal component analysis with oblique rotation, identified two mood factors with eigenvalue >1 [41]. Ratings on the items ‘enthusiastic’, ‘happy’, ‘cheerful’, ‘strong’, and ‘satisfied’ –weighted for factor loadings- were averaged to form a PA scale. Ratings on the items ‘anxious’, ‘irritated’, ‘harried’, ‘tense’, ‘lonely’, ‘gloomy’, ‘guilty’, ‘edgy’, ‘insecure’, and ‘tired’ were averaged to form an NA scale.

#### Depressive symptoms

Clinical outcome was measured with the HDRS [38]. A trained research assistant conducted the assessments during telephone interviews. A random sample of 16 interviews was simultaneously rated by an experienced psychiatrist in order to check the quality of assessments and avoid ‘drift’.

### Statistical analysis

In order to categorize treatment response, change in HDRS scores was dichotomized. Patients with the 50% largest decrease in HDRS score were classified as responders, and the remaining 50% were classified as non-responders. Response was assessed eight weeks after the start of treatment. We created one group status variable defining healthy controls, non-responders, and responders. A second group status variable was created including separate categories for healthy controls, first episode non-responders, first episode responders, recurrent episode non-responders, and recurrent episode responders. Centering and multilevel regression analyses were performed as in study 1. We examined by multilevel regression analysis whether momentary PA level at (*t-1*) (independent variable) predicted momentary PA level at (*t*) (dependent variable). An interaction was fitted between PA at (*t-1*) and the three level group status variable (healthy controls, non-responders, and responders), in order to examine whether the transfer of PA from (*t-1*) to (*t*) differed between healthy controls, patients who did, and patients who did not respond to treatment (Figure 1). Furthermore, we analyzed whether momentary transfer of PA differed between patients with a first episode and patients with recurrent depression by fitting an interaction between PA at (*t-1*) and the five-level group status variable (healthy controls, first episode non-responders, first episode responders, recurrent episode non-responders, and recurrent episode responders). As in study 1, analyses were corrected for level of NA and all effect sizes were standardized.

### Results

Level of PA at beep (*t-1*) significantly predicted level of PA at the following beep (β=.446, SE=.015, *p*<.001). Non-responders showed less transfer of momentary PA at baseline than healthy controls (β interaction=-.090, SE=.043, *p*<.05). Responders showed greater transfer of momentary PA when compared to non-responders (β interaction=.105, SE=.048, *p*<.05), and did not differ from healthy controls.

**Figure 3:**
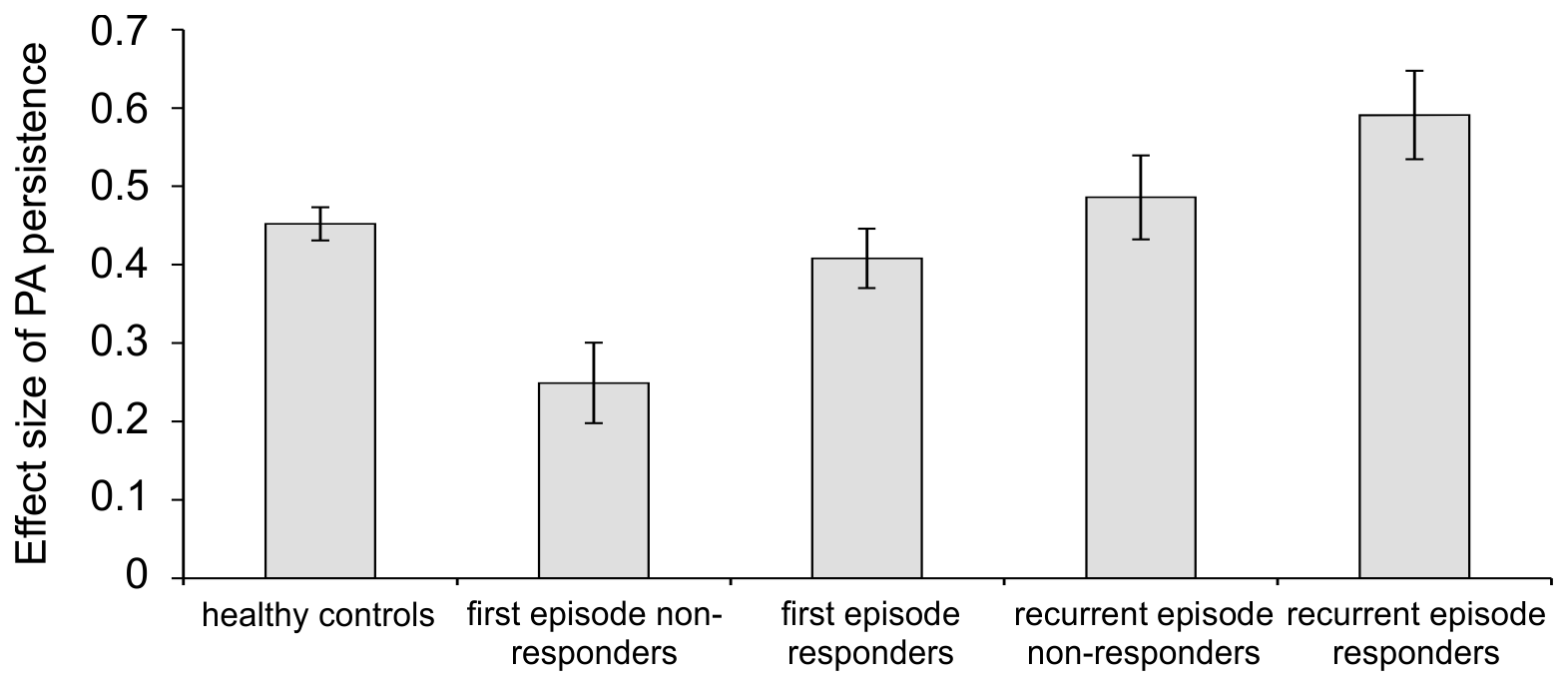
Degree of momentary PA persistence as a function of treatment response, first/recurrent episode and healthy control status. Bars represent the association between PA at (*t-1*) and PA at (*t*) (level of momentary transfer of positive affect (PA) from (*t-1*) to (*t*) approximately 90 minutes, or level of PA persistence), stratified by treatment response and first episode/recurrent episode subgroups. Error bars represent one standard error of the mean.

Examining the effect of first episode versus recurrent depression status on transfer of momentary PA revealed the following results (Table 2): Compared to healthy controls, first episode non-responders showed significantly less transfer of momentary PA, while recurrent episode responders showed significantly greater transfer of momentary PA. Other groups did not significantly differ from healthy controls. Compared to first episode non-responders, all other groups of depressed patients had significantly greater transfer of momentary PA. Standardized effect sizes per group are displayed in Figure 3.

**Table 2:** PA persistence (association between PA(*t-1*) and PA(*t*)) in study 2, stratified by responder/non-responder and first/recurrent subgroup.

**Reference group**		β	SE	*p*-value
Healthy controls	First episode non-responders	-.203	.055	<.001
	Recurrent episode non-responders	.034	.057	.55
	First episode responders	-.044	.043	.31
	Recurrent episode responders	.139	.060	.02
First episode non-responders	Recurrent episode non-responders	.237	.074	.001
	First episode responders	.159	.063	.01
	Recurrent episode responders	.342	.075	<.001
Recurrent episode non-responders	First episode responders	-.078	.065	.23
	Recurrent episode responders	.105	.077	.17
First episode responders	Recurrent episode responders	.183	.067	.007

β = effect size of PA(*t-1*) on PA(*t*), moderated by group status; SE = standard error.

## Study 3

### Participants

A sample of 83 patients was recruited in eight primary care practices in the Netherlands to participate in a randomized controlled trial (RCT) examining the effects of imipramine compared to placebo on daily quality of life. Inclusion criteria were a DSM-IV diagnosis of current major depressive disorder, age between 18 and 65 years, a baseline score of ≥18 on the 17-item HDRS, and a score of ≥4 on the Clinical Global Impression scale (CGI) [42]. Patients with major somatic complaints or using psychotropic medications, except for the occasional use of temazepam, were excluded. Furthermore, 29 healthy participants were recruited in primary care practices to provide normal reference values for ESM measures. This comparison group was matched to the depressed group in terms of age, sex distribution, and socioeconomic status (for details, see [43]).

In the patient group 12 were excluded because they did not meet the inclusion criteria, six patients provided less than 30% valid ESM reports, one patient had a missing baseline HDRS score, and 14 patients had missing follow-up HDRS scores. Of 29 screened controls, six were excluded due to lifetime depression or current psychiatric illness, and two participants provided less than 30% valid ESM reports. The mean number of completed ESM reports was 47.1 (SD 7.6; range 21-59). The final sample consisted of 71 participants: 50 patients and 21 healthy controls (Table 3). There were 23 patients in the imipramine group, of which 14 were classified as responders to treatment. There were 27 patients in the placebo group, of which 9 were classified as responders to treatment.

**Table 3:** Demographic data for depressed (imipramine/placebo treatment) and healthy participants.

	Depressed (imipramine treatment)	Depressed (placebo treatment)	Healthy controls
N	23	27	21
%women	78	74	76
Mean age (SD)	42.6 (9.7)	42.3 (8.7)	42.0 (11.2)
- range	26-59	25-58	18-62
% married	86	59	83
% secondary education	65	73	64
% college/university	13	13	6
% employed	65	29	51
% homemaker	31	25	18
% in school	0	11	6
% unfitted for work/unemployed	5	22	6

SD = standard deviation.

### Study design

In a RCT procedure as described in [44], all participants (including the control group) underwent ESM at baseline for six consecutive days. During the initial baseline week, participants received no treatment in any form. Thereafter, patients were randomly assigned to twice daily, double-blind, 6-week treatment with either imipramine (starting dose of 50 mg/day, increased to 200 mg/day over the first week of treatment) or a placebo (starting with one capsule per day, increased to four capsules over the first week of treatment). In order to assess the course of depression, the HDRS was administered at baseline and at week six. The healthy controls served as reference group and were not randomized to any treatment.

### Measurements

#### Affective states

The ESM affect items were selected according to the procedure described in study 1. Factor analysis, using principal component analysis with oblique rotation, identified two mood factors with eigenvalue >1 [44]. Ratings on the items ‘content’, ‘enthusiastic’, ‘cheerful’, ‘strong’, ‘energetic’, ‘alert’, ‘calm’, and ‘happy’ –weighted for factor loadings- were averaged to form a PA scale. Ratings on the items ‘depressed’, ‘lonely’, ‘irritable’, ‘insecure’, ‘harried’, ‘anxious’, ‘guilty’, ‘hostile’, and ‘tensed’ were averaged to form an NA scale.

#### Depressive symptoms

The 17-item HDRS was administered by the treating general practitioner (GP). All participating GPs had completed a standardized training for the HDRS procedure.

### Statistical analysis

Responders and non-responders were classified as in study 2. Response was assessed six weeks after the start of treatment. We created one group status variable defining healthy controls, patient non-responders, and patient responders. A second group status variable was created including separate categories for healthy controls, imipramine responders, imipramine non-responders, placebo responders, and placebo non-responders. Centering and multilevel regression analyses were performed as in studies 1 and 2. We examined whether momentary PA level at (*t-1*) (independent variable) predicted momentary PA level at (*t*) (dependent variable). An interaction was fitted between PA at (*t-1*) and the three-level group status variable (healthy controls, non-responders, responders), in order to examine whether the transfer of PA from (*t-1*) to (*t*) differed between healthy controls, patients who did, and patients who did not respond to treatment (Figure 1). Furthermore, we analyzed whether momentary transfer of PA differed between patients who received imipramine and patients who received a placebo by fitting an interaction between PA at (*t-1*) and the five-level group status variable (healthy controls, imipramine responders, imipramine non-responders, placebo responders, and placebo non-responders). As in the previous two studies, analyses were corrected for level of NA and all effect sizes were standardized.

### Results

Level of PA at any beep significantly predicted level of PA at the next beep (β=.517, SE=.016, *p*<.001). This association was significantly different for responders, non-responders, and healthy controls. More specifically, non-responders showed less momentary transfer of PA at baseline than healthy controls (β interaction=-.143, SE=.042, *p*<.001). Responders, however, had higher levels of momentary transfer of PA than healthy controls (β interaction=.118, SE=.043, *p*<.01).

**Figure 4:**
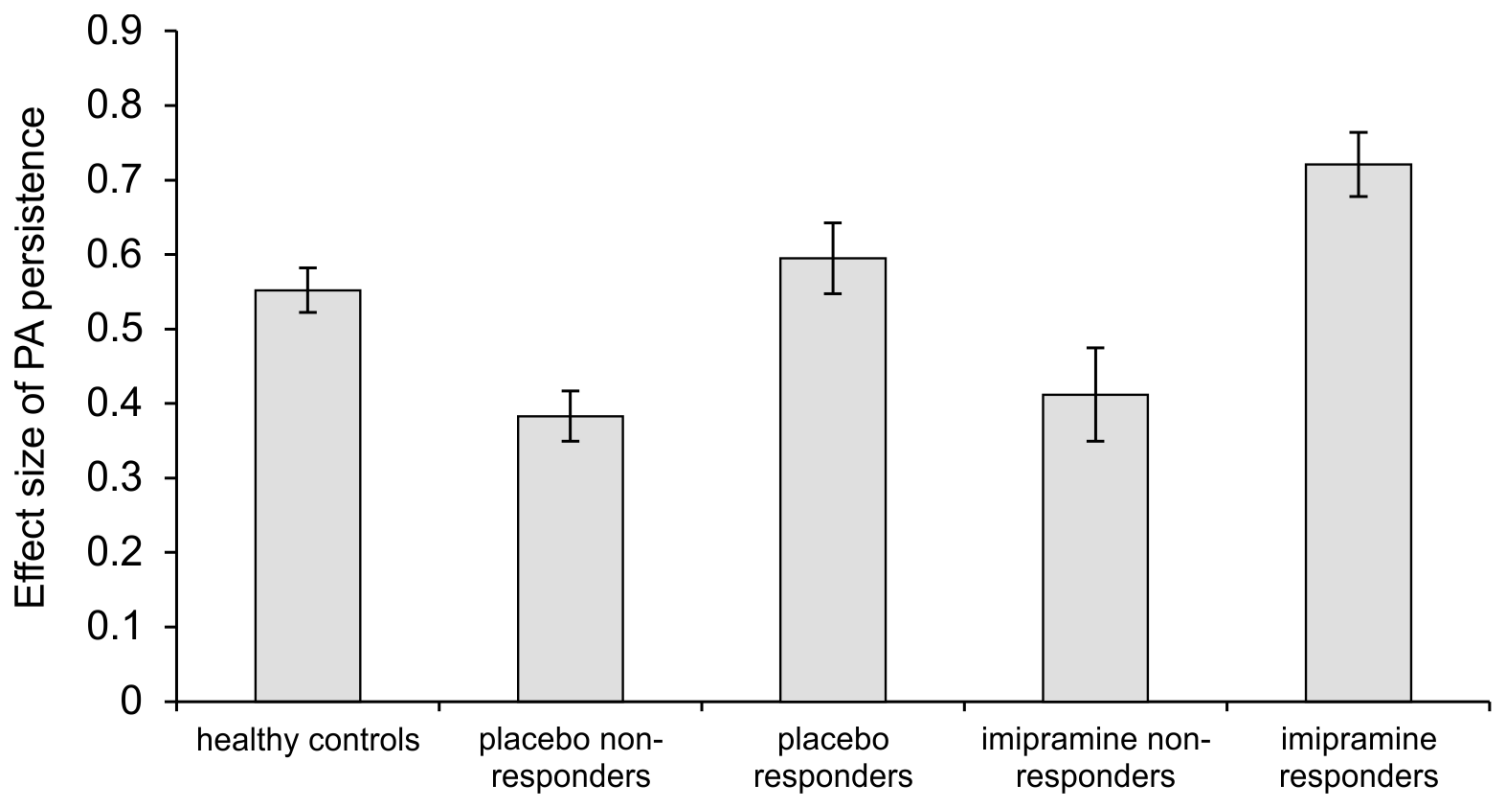
Degree of momentary PA persistence as a function of treatment response, placebo/imipramine and healthy control treatment status. Bars represent the association between PA at (*t-1*) and PA at (*t*) (level of momentary transfer of positive affect (PA) from (*t-1*) to (*t*) approximately 90 minutes, or level of PA persistence), stratified by treatment response and placebo/imipramine subgroups. Error bars represent one standard error of the mean.

Examining the effect of type of treatment (imipramine/placebo) revealed that responders in the imipramine group had significantly greater momentary transfer of PA at baseline than placebo responders or imipramine and placebo non-responders (Table 4). Standardized effect sizes per group are displayed in Figure 4.

**Table 4:** PA persistence (association between PA(*t-1*) and PA(*t*)) in study 3, stratified by responder/non-responder and placebo/imipramine subgroup.

**Reference group**		β	SE	*p*-value
Healthy controls	Placebo non-responders	-.168	.044	<.001
	Imipramine non-responders	-.140	.069	.04
	Placebo responders	.043	.056	.44
	Imipramine responders	.170	.052	.001
Placebo non-responders	Imipramine non-responders	.028	.070	.69
	Placebo responders	.212	.057	<.001
	Imipramine responders	.338	.053	<.001
Placebo responders	Imipramine non-responders	-.183	.078	.02
	Imipramine responders	.126	.063	.05
Imipramine non-responders	Imipramine responders	.310	.075	<.001

β = effect size of PA(*t-1*) on PA(*t*), moderated by group status; SE = standard error.

## Discussion

This is the first study to show that the ability to savor PA over time in daily life predicts future course of depressive symptoms in both healthy and depressed individuals. This effect was replicated, using the same methodology, across three independent samples. We found that individuals from a general population sample with a higher baseline ability to savor PA in daily life had a lower level of depressive symptoms at follow-up. In the first clinical sample, baseline transfer of PA over time predicted response after eight weeks to combined treatment with antidepressants and psychotherapy. This effect was more pronounced in patients with a history of depressive episodes than in patients who were depressed for the first time. Finally, the results from the second clinical sample revealed that baseline moment-to-moment transfer of PA over time predicted treatment response after six weeks. Moreover, allocation to imipramine rather than placebo was related to a stronger association between moment-to-moment transfer of PA and treatment response.

Consistent with prior research [2,3,6-10], our results underscore the protective properties of PA against depression. This study suggests that this protective mechanism is partly mediated by the ability to savor PA in daily life.

### Relation to previous findings regarding persistence of positive affect

The days during which the focus of depression research was solely on negative emotions have long passed. Contemporary research seeks to determine to what extent positive emotions are a factor in prevention and treatment of depressive illness and whether they can predict the future course of the disorder [2,3,6-10]. Although several studies have examined PA reactivity to pleasant situations [2,8,9,19,22], PA *persistence* remains understudied.

The current findings are compatible with work presented by Heller and colleagues [23], who showed that depressed patients have difficulty sustaining activity in brain areas responsible for PA and reward. It is attractive to speculate that these findings represent the neurobiological correlates of lower level moment-to-moment transfer of PA in real life in depressed patients, as reported in the current study. A recent ESM study by Thompson and colleagues [26], however, found no difference in momentary PA persistence between depressed individuals and healthy controls. This contrasts with the current findings, which indicated that generally lower PA persistence was encountered in depressed patients as compared to healthy controls. One potential difference between Thompson and colleagues’ and our study to explain the discrepancy in findings may be found in the group characteristics that were compared. While Thompson and colleagues compared depressed patients with healthy controls, our study mainly focuses on the difference in response to treatment within the patient group as compared to healthy controls. That is, we did not compare healthy individuals with depressed patients in general, but with responders and non-responders, respectively. Finally, a study by Kuppens and colleagues [24] found that persistence of happy feelings was related to *higher* levels of current and future [25] depressive symptoms. In their experiment, adolescents and their families were instructed to talk about difficult family situations for 20 minutes while they were being videotaped. The adolescents’ verbal and non-verbal behavior during the interaction was rated, and happy, angry and dysphoric behaviors were derived. The emotional behavior of current and future depressed adolescents appeared to be more resistant to changing circumstances. That is, they showed greater persistence of positive as well as negative emotional behavior than healthy participants. It is possible that these contrasting findings reflect important differences in study design and methodology. While we measured savoring PA in daily life at random moments in the day, Kuppens and colleagues measured the persistence over time in emotional *responses* to a certain situation in the laboratory. A fundamentally different concept may therefore have been measured. Kuppens and colleagues explain their findings in that a stronger persistence of PA or NA seems to indicate an affective resistance to change, reflecting impaired adjustment to the environment (*emotional inertia*) or inflexibility [25]. The findings in the current study on moment-to-moment transfer of PA pertained to measurements at a different time scale (measurements were on average 90 minutes apart over several days and were not assessed minute-to-minute in a 20 minute lab experiment) and do not reflect persistence of an emotional response to a certain situation. Since measurements in the current study were further apart than in the study of Kuppens and colleagues [24,25], it is additionally possible that in the current study a stronger persistence of PA reflected the ability to use PA to create new PA in the near future. All in all, the above differences in results and methodology highlight the importance of within-study replication with constant methodology.

### Savoring positive affect as compensatory mechanism

The current study showed that increased transfer of PA over time predicted better follow-up response to treatment in patients. Particularly surprising was the fact that persistence of PA over time was not only higher in responding versus non-responding depressed patients, but also in responding depressed patients versus healthy controls. Notably, increased levels of PA persistence in depressed patient responders –compared to controls- were found in both patient samples. Better-than-normal ability to hold on to PA may be required for depressed patients in order to recover. The differences in depressed patients compared to healthy individuals are often viewed upon as reflecting risk. However, the current findings suggest that these specific alterations indicate a better than average ability to savor PA. This extraordinary quality, or resilience trait, may reflect a compensatory mechanism in that increased ability to hold on to PA is required to counteract the increased presence of NA in these individuals. A speculation is that only in case of strong compensatory mechanisms, which can restore emotional balance, patients will be able to recover.

Although responders generally appeared to be better at savoring PA than non-responders, this effect was more pronounced in patients with a history of depressive episodes compared to patients who were depressed for the first time. This interesting finding raises the possibility that, due to the recurrence of their depression, a learning process may have taken place. Over time, patients with recurrent episodes may have acquired ways to savor their PA, thereby aiding the process of recovery [41]. However, these mechanisms need to be examined in more depth before any firm conclusions can be drawn.

The replication of the finding of higher PA persistence in depressed patients who respond to treatment compared to healthy controls in two independent samples underscores our findings, and indicates that specific groups of depressed patients paradoxically may possess higher-than-average ability to preserve PA that can facilitate recovery.

### Medication and savoring positive affect

Patients who responded to imipramine treatment showed higher levels of PA persistence than responders who were treated with a placebo. There are different ways to interpret this finding. One potential interpretation is that antidepressant medication acted synergistically with baseline ability to savor PA in bringing about a reduction in depressive symptoms. This interpretation suggests that medication may give an extra boost to the favorable effect of baseline PA persistence on follow-up outcome. An alternative interpretation is that for those using imipramine a higher baseline PA persistence is required in order to become a responder than for those using a placebo. Although counterintuitive, there have been previous studies suggesting that some antidepressants are associated with ‘emotional blunting’ [45,46], reduced reward experience [47], and diminished processing of not only negative but also positive stimuli [20]. In other words, the antidepressant may not only diminish negative, but positive emotional experience as well. Also, recent studies have shown that PA is important in facilitating recovery from depression [7-10]. With respect to the current findings this may suggest that patients who receive antidepressants may *need* to be better at savoring PA in order to counteract the PA-dampening effects of medication. This interpretation is speculative, since the above studies all employed SSRIs, whereas the patients in our study received a tricyclic antidepressant (TCA). Further exploration of the long-term underlying psychological effects of antidepressants is needed in order to shed more light on the influence of medication on PA.

### Limitations

A limitation regarding study 1 is that the sample consisted of female participants only. Consequently, our findings may not generalize to men. A limitation regarding the patient samples (studies 2 and 3) is that follow-up depressive symptoms were not assessed at the exact same time point following baseline measurements. That is, in study 2, depressive symptoms were assessed eight weeks following the start of the treatment, whereas in study 3, they were assessed after six weeks. It should be noted as well that patients in study 2 were treated with a combination of pharmacotherapy and supportive psychotherapy, whereas in study 3 they received pharmacotherapy only. Also, whereas in study 2 pharmacotherapy consisted mainly of serotonergic antidepressants, in study 3 patients received TCAs. Despite these differences in methodology, similar results were reported regarding the effect of PA persistence on follow-up responder status in both studies. Finally, a general limitation is that there are some differences in the adjectives that were used to conceptualize PA and NA in the three studies. This was a consequence of the fact that we used existing ESM samples to test our hypothesis. Slightly different choices for one item or another were made by the researchers who conducted the original studies. However, most items were identical and for each study the items loaded on one factor of PA.

## Conclusion

The present findings indicate that healthy individuals with greater PA persistence have a significantly smaller probability of developing increased levels of depressive symptoms, and that depressed patients are more likely to respond to their treatment if they have greater PA persistence at baseline. This is the first study that, consistently and in three different samples, indicates that PA persistence, as measured in the flow of daily life, predicts future course of depressive symptoms. 
